# Supplementary material for: MCT1-governed pyruvate metabolism is essential for antibody class-switch recombination through H3K27 acetylation
Source: Nat Commun. 2024 Jan 2;15:163. doi: 10.1038/s41467-023-44540-0 (PMC10762154; doi:10.1038/s41467-023-44540-0)
Supplement: Supplementary file 1 — Supplementary Information [file 41467_2023_44540_MOESM1_ESM.pdf]

Supporting Information

MCT1-governed pyruvate metabolism is essential for antibody class-switch recombination through H3K27 acetylation

Wenna Chi[1, 2#], Na Kang[3, 4#], Linlin Sheng[1#], Sichen Liu[3#], Lei Tao[1,2], Xizhi Cao[1], Ye Liu [1], Can Zhu[3], Yuming Zhang[1], Bolong Wu[1], Ruiqun Chen[1], Lili Cheng[1], Jing Wang[3], Xiaolin Sun[5, 6], Xiaohui Liu[7], Haiteng Deng[7], Jinliang Yang[2], Zhanguo Li[4, 5, 6],

Wanli Liu[3, 4*], Ligong Chen [1, 2*]

1. School of Pharmaceutical Sciences, Key Laboratory of Bioorganic Phosphorus Chemistry and Chemical Biology (Ministry of Education), Tsinghua University, Beijing 100084, China;
2. Collaborative Innovation Center for Biotherapy, State Key Laboratory of Biotherapy and Cancer Center, West China Hospital, West China Medical School, Sichuan University, Chengdu 610065, China;
3. State Key Laboratory of Membrane Biology, School of Life Sciences, Institute for Immunology, China Ministry of Education Key Laboratory of Protein Sciences, Beijing Key Lab for Immunological Research on Chronic Diseases, Tsinghua University, Beijing, 100084, China;
4. Tsinghua-Peking Center for Life Sciences, China;
5. Department of Rheumatology and Immunology, Peking University People's Hospital, Beijing 100044, China;
6. Beijing Key Laboratory for Rheumatism Mechanism and Immune Diagnosis (BZ0135), Beijing 100044, China;
7. National Center for Protein Science, School of Life Sciences, Tsinghua University, Beijing 100084, China.

# These authors equally contribute to the project.
* To whom correspondence should be addressed:
Ligong Chen, Professor, School of Pharmaceutical Sciences, Tsinghua University, Beijing, CHINA 100084. Email: ligongchen@tsinghua.edu.cn
Wanli Liu, Professor, Institute for Immunology, School of Life Sciences, Tsinghua University, Beijing, CHINA 100084. Email: liulab@tsinghua.edu.cn

This PDF file includes: Supplementary Fig.1 to Supplementary Fig.15

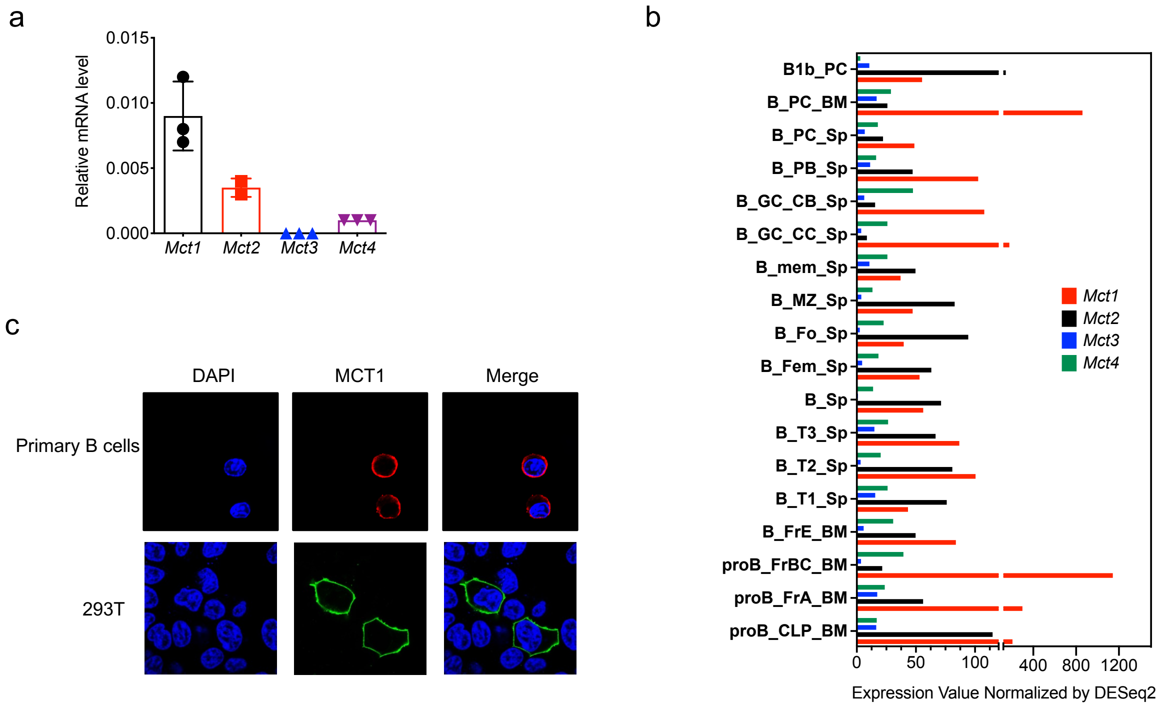

Supplementary Fig.1 *Mct1* is highly transcribed on B cells. a The mRNA levels of *Mct1-4* in the primary B cells stimulated with LPS and IL-4 for 2 days by qPCR, n = 3 biological replicates. **b** Analyze the expression of *Mct1-4* in B cells through the RNA-seq database from ImmGen. **c** Immunofluorescence analysis of MCT1 sub-localization of primary B cells (top panel) and 293T cells (bottom panel). MCT1 protein are red (top panel) or green (bottom panel) and nuclei are blue (DAPI), n = 3 biological replicates. Scale Bar, 10 μm. Data are presented as mean ± SEM, unpaired two-tailed t-test, * $p < 0.05$, ** $p < 0.01$, n.s, not significant.

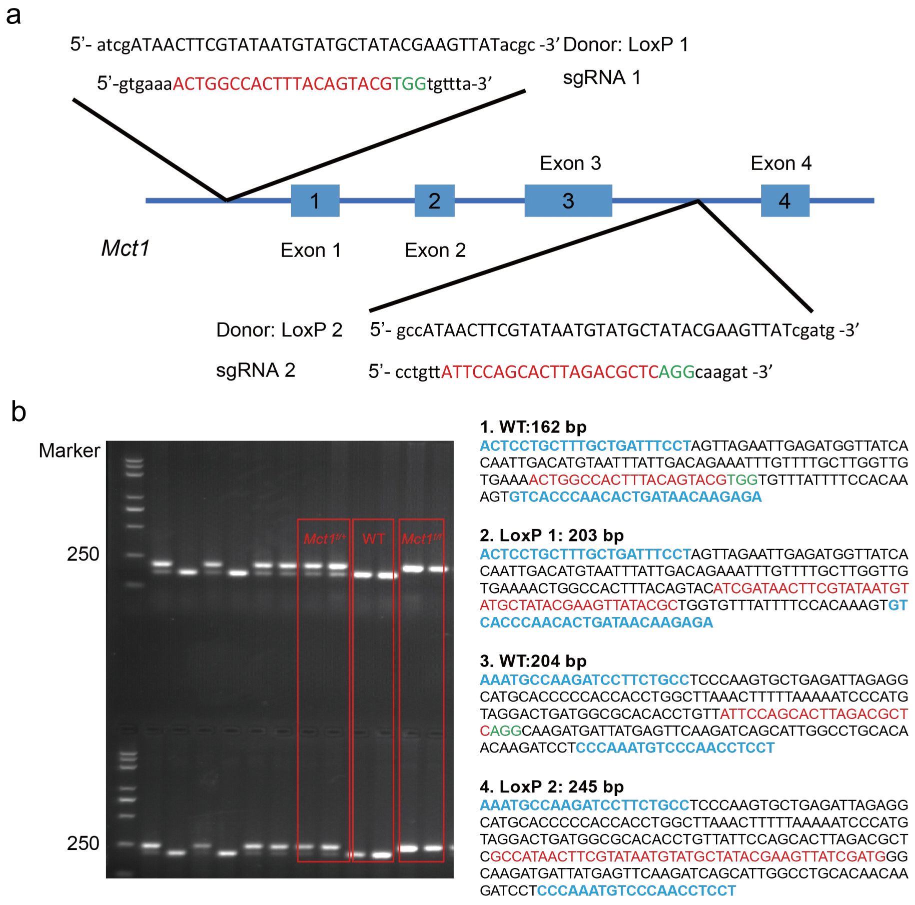

Supplementary Fig.2 The *Mct1*$^{f/f}$ mice were obtained with CRISPR-Cas9 technology.
a The schematic diagram of the sgRNA/donor sites at *Mct1* allele. The sgRNAs sequences are indicated in red, the protospacer-adjacent motif (PAM) in green and the LoxP donor sequences in black capital letters. **b** PCR identification analysis of targeted alleles. Expected fragment size: 1-WT = 162 bp, 2-LoxP 1 = 203 bp, 3-WT = 204 bp, and 4-LoxP 2 = 245 bp. The PCR primers are shown in blue.

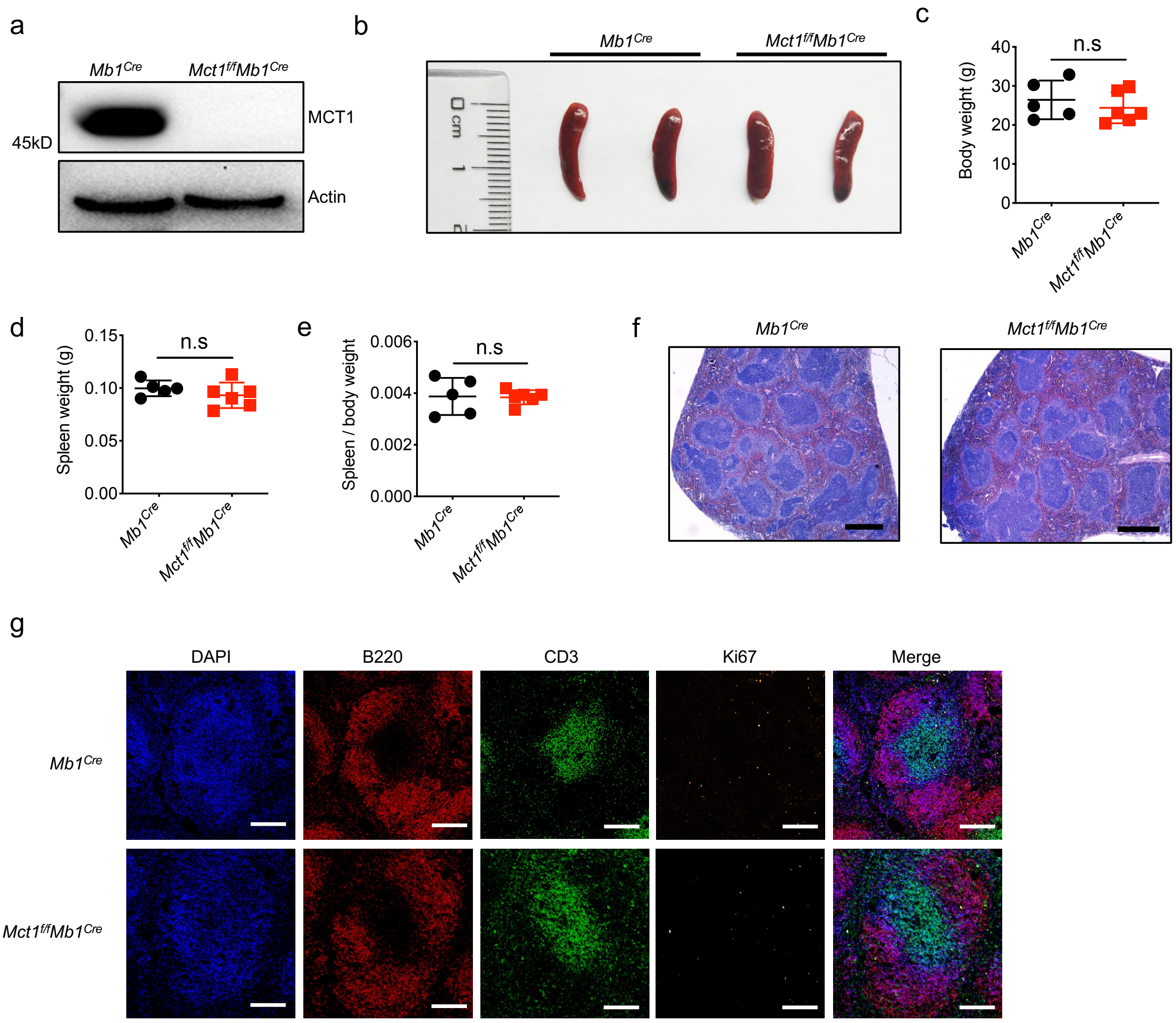

Supplementary Fig.3 *Mct1* deficiency does not alter spleen morphology and weight. a The protein level of MCT1 in the activated B cells with LPS and IL-4-treated for 3 days was assessed by western blotting,n = 3 biological replicates. **b-g** The *Mb1*^{Cre} and *Mct1*$^{f/f}$*Mb1*^{Cre} mice without immunization, the size (**b**) and weight (**c**) of spleen, body weight (**d**) and the ratio of spleen and body (**e**) were shown, *Mb1*^{Cre} (n = 5 biological replicates) and *Mct1*$^{f/f}$*Mb1*^{Cre} (n = 6 biological replicates). The morphology of the red and white pulp of the spleen were analyzed by H&E. Scale Bar, 250 μm (**f**). Proliferative B cells were detected by immunofluorescence (IF) with Ki67 (proliferative cells marker), CD3 (Negative, T cells), and B220 (Positive, B cells). DAPI is nuclear marker. Scale Bar, 50 μm (**g**). Data (**b, f, g**) are representative one of three independent experiments. Data are presented as mean ± SEM of 5-6 mice, unpaired two-tailed t-test, n.s, not significant.

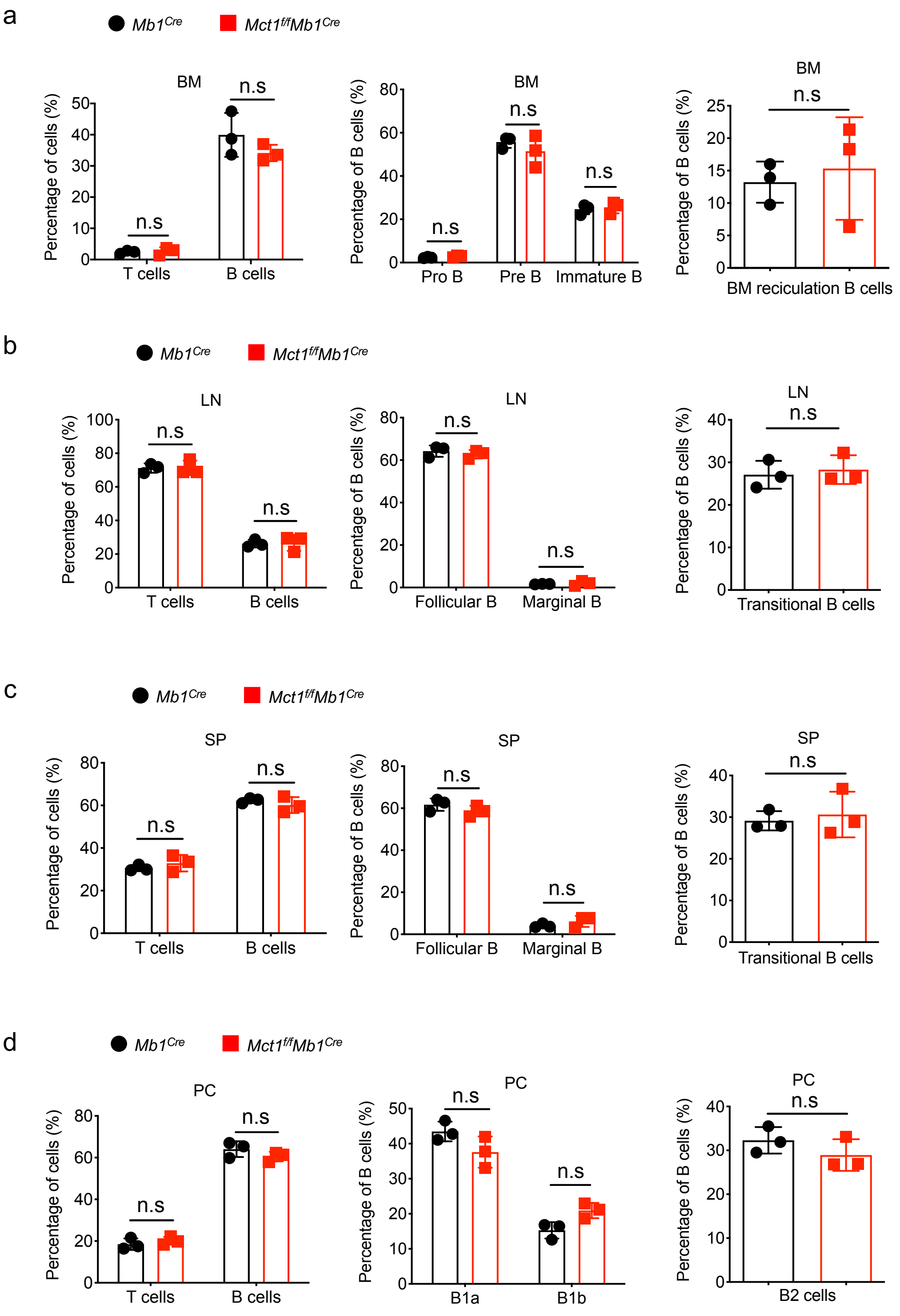

Supplementary Fig.4 *Mct1* deficiency does not impair the B cells development. a-d Flow cytometry analysis of the frequencies of T cells, B cells, immature B cells, pro-B, pre-B, recirculating mature B cells, follicular B cells, marginal B cells, transitional B cells, B1a, B1b and B2 cells in bone marrow (BM) (**a**), lymph node (LN) (**b**), spleen (SP) (**c**), or peritoneal cavity (PC) (**d**), respectively, n = 3 biological replicates. Data are presented as mean ± SEM of 3 mice, unpaired two-tailed t-test, n.s, not significant.

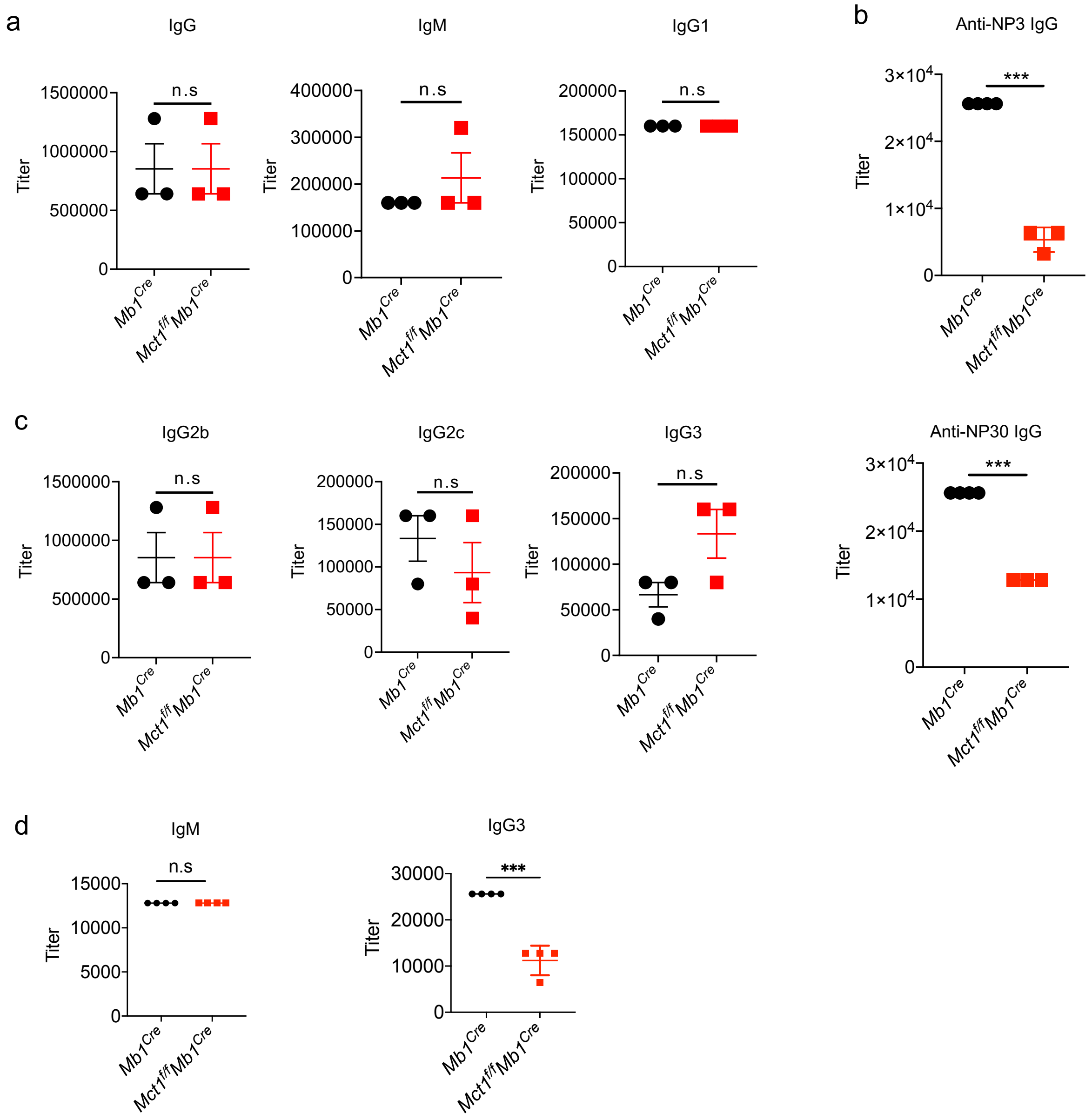

Supplementary Fig.5 *Mct1* deficiency does not affect the serum innate antibodies homeostasis, but affects antibodies isotypes response afer immunization with NP_{33}-KLH. a Titer of innate antibody IgM, IgG, IgG1, IgG2b, IgG2c, or IgG3 isotype were detected by ELISA, n = 3 biological replicates. **b** The titers of IgG responding to Anti-NP3 or Anti-NP30 from *Mb1*^{Cre} (n = 4 biological replicates) and *Mct1*$^{f/f}$*Mb1*^{Cre} (n = 3 biological replicates) mice upon immunization (i.p.) with NP_{33}-KLH at week 2 were detected by ELISA. **c** Antibody titers of IgG1, IgG2b, IgG2c, or IgG3 isotype from *Mb1*^{Cre} (n = 3 biological replicates) and *Mct1*$^{f/f}$*Mb1*^{Cre} (n = 3 biological replicates) upon immunization (Intraperitoneal injection, i.p.) with NP_{33}-KLH at week 2 were detected by ELISA. D. Antibody titers of IgG3 and IgM isotype from *Mb1*^{Cre} (n = 4 biological replicates) and *Mct1*$^{f/f}$*Mb1*^{Cre} (n = 4 biological replicates) upon immunization (i.p.) with NP-Ficoll at week 2 were detected by ELISA. Data are presented as mean ± SEM of 3-4 mice, unpaired two-tailed t-test, * $p < 0.05$, ** $p < 0.01$, n.s, not significant.

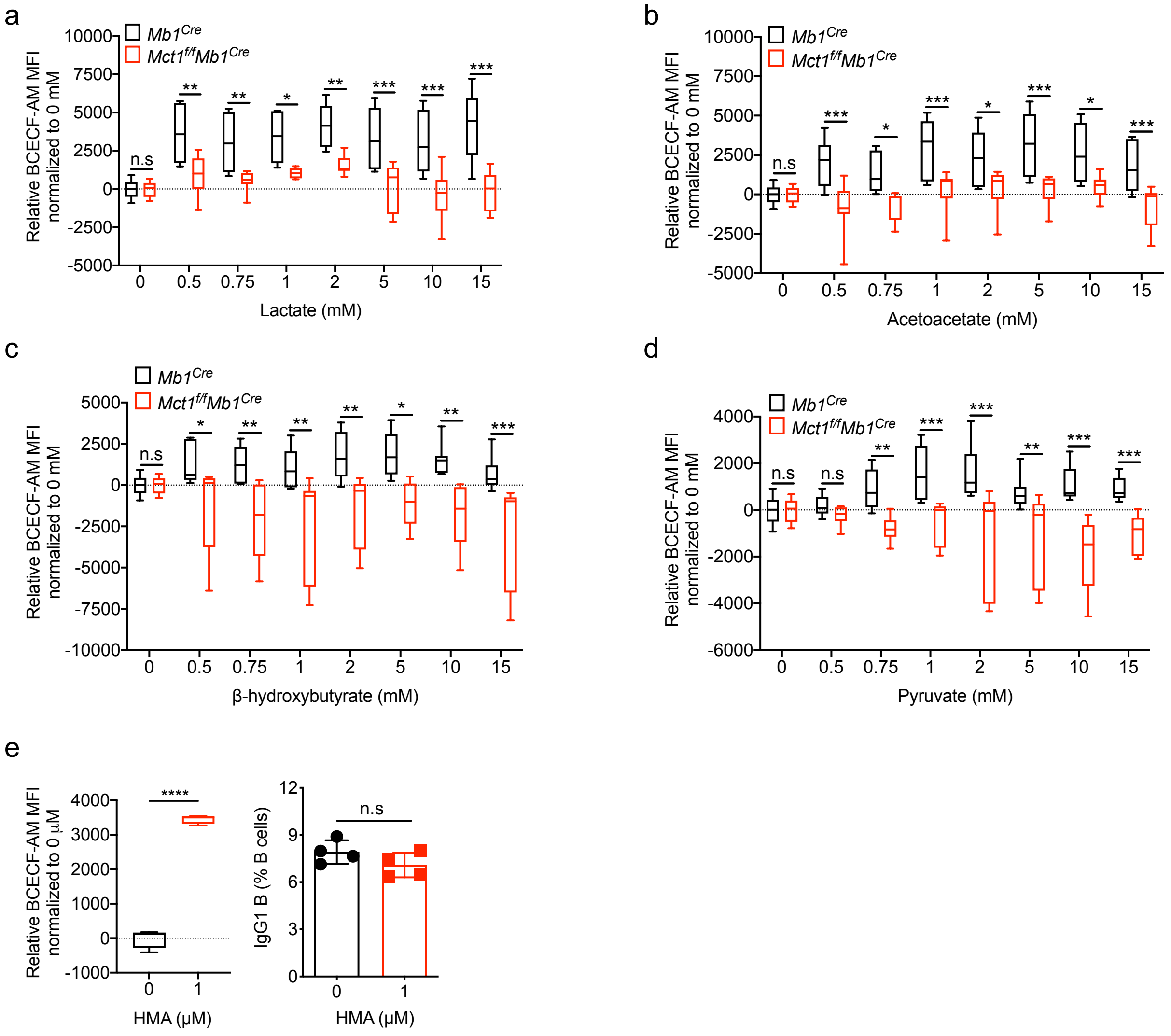

Supplementary Fig.6 *Mct1* deficiency affected the CSR in a pH-independent manner.
a Naïve B cells isolated from *Mb1*^{Cre} and *Mct1*$^{f/f}$*Mb1*^{Cre} mice were cultured with various concentration of lactate (**a**), acetoacetate (**b**), β-hydroxybutyrate (**c**) and pyruvate (**d**), then pH was measured (n = 3 biological replicates). **e** Naïve B cells isolated from *Mb1*^{Cre} and *Mct1*$^{f/f}$*Mb1*^{Cre} mice were co-stimulated with LPS and IL-4 and added with or without HMA (5-N,N-hexamethylene amiloride, an inhibitor of the Na^+/H^+ exchanger) for 3 days (n = 4 biological replicates). Data are presented as mean ± SEM of 3-4 mice, unpaired two-tailed t-test, * $p < 0.05$, ** $p < 0.01$, n.s, not significant.

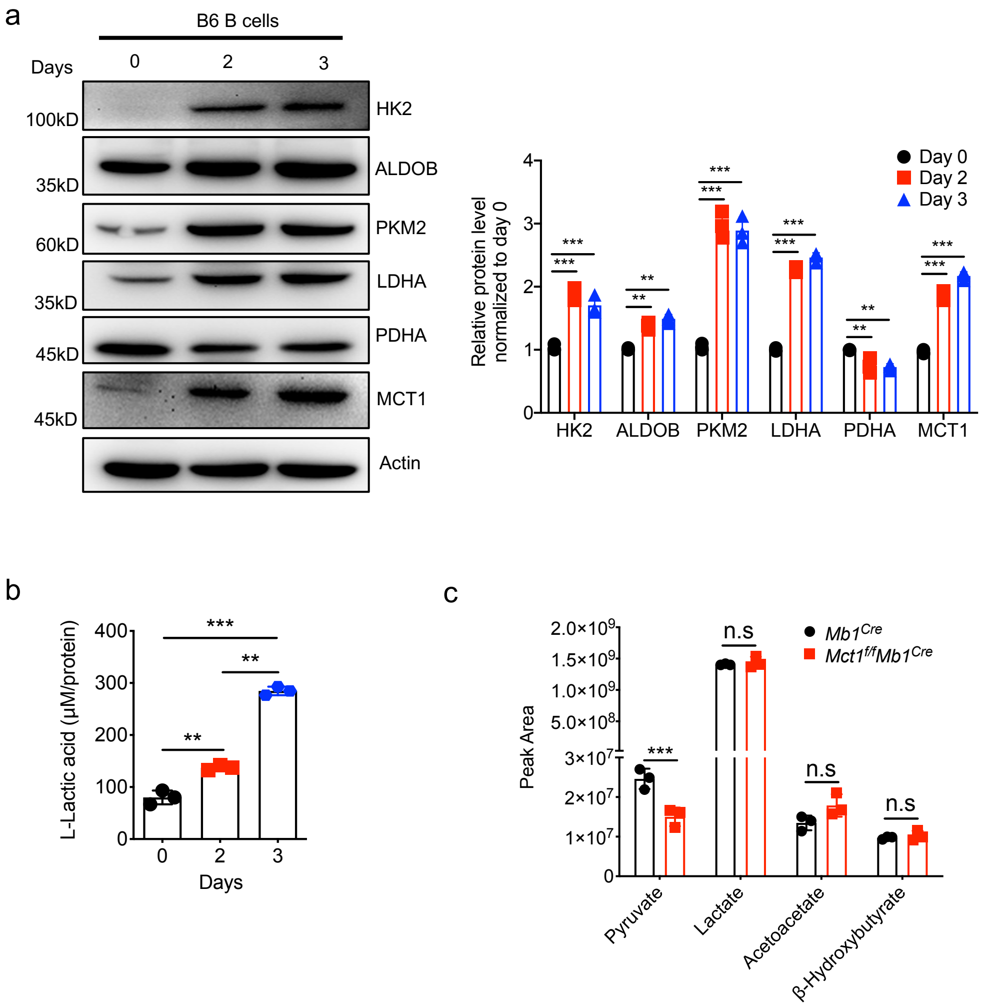

Supplementary Fig.7 LPS and IL-4 treatment increases glucose utilization in primary B cells *in vitro*. a Glycolysis enzyme expression in the activated B cells treated with LPS and IL-4 for 2 or 3 days was assessed by western blotting (n = 3 biological replicates). **b** Naïve B cells isolated from C57BL6J (B6) mice were stimulated with LPS and IL-4 for 2 or 3 days. The lactate concentrations of naïve and activated B cells were detected by L-lactate assay kit (Elabscience) (n = 3 biological replicates). **c** Naïve B cells isolated from $Mb1^{Cre}$ and $Mct1^{f/f}Mb1^{Cre}$ mice were stimulated with LPS and IL-4 for 3 days. The relative level of pyruvate, lactate, acetoacetate and β-hydroxybutyrate in intracellular was determined by metabonomics (n = 3 biological replicates). Data are presented as mean ± SD, two-way ANOVA followed by Sidak's multiple-comparisons test, $^{**}p < 0.01$, $^{***}p < 0.001$, n.s, not significant. Source data are provided as a Source Data file.

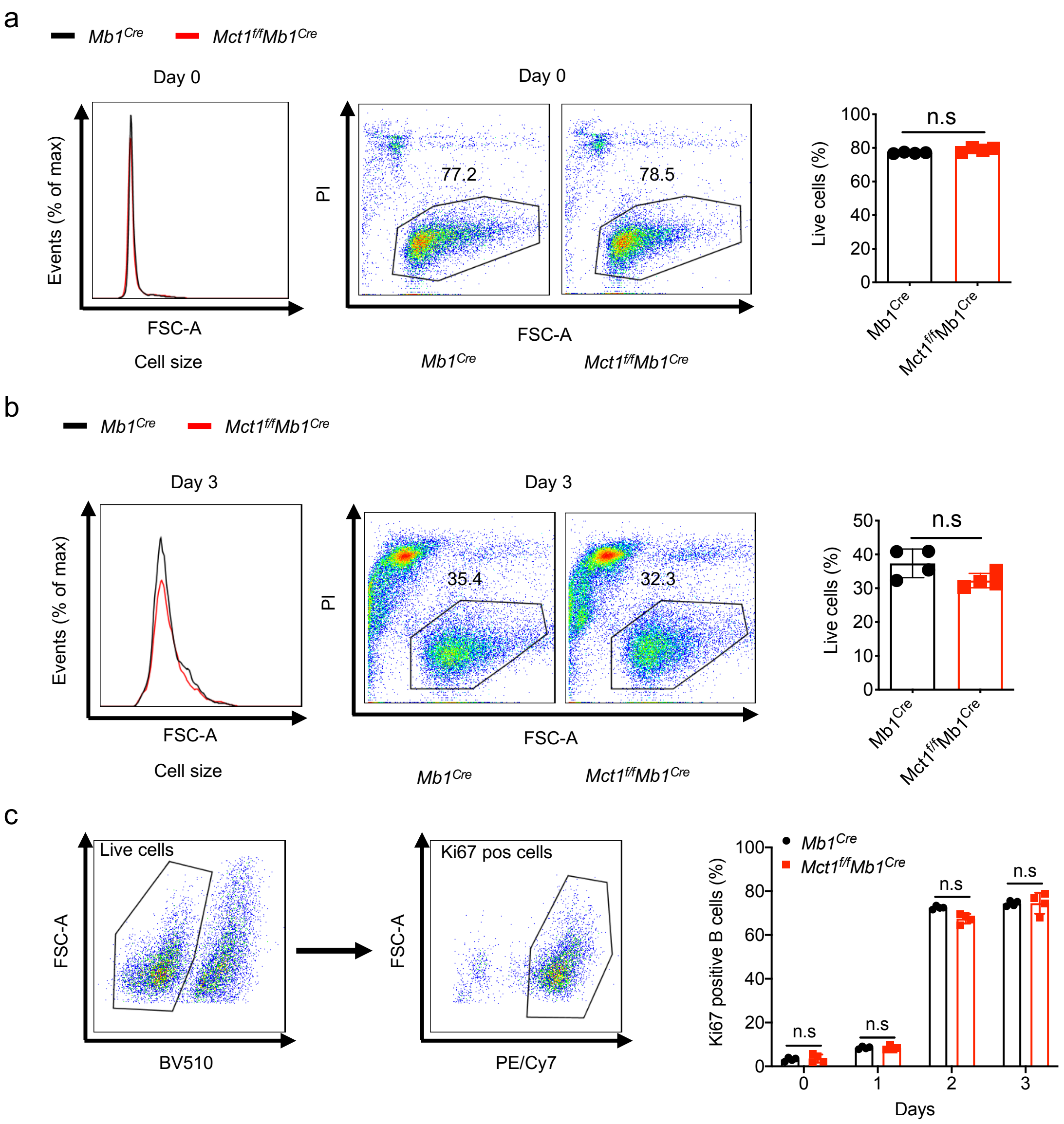

Supplementary Fig.8 MCT1 is nonessential for B cells proliferation and survival *in vitro*. **a** Naïve B cells isolated from *Mb1*^{Cre} and *Mct1*$^{f/f}$*Mb1*^{Cre} mice. Cell size (left) and PI staining (center) of B cells were analyzed by flow cytometry. Cumulative data of live cells was shown in right, n = 4 biological replicates. **b** Naïve B cells isolated from *Mb1*^{Cre} and *Mct1*$^{f/f}$*Mb1*^{Cre} mice were stimulated with LPS and IL-4 for 3 days. Cell size (left) and PI staining (center) of B cells were analyzed by flow cytometry. Cumulative data of live cells was shown in right, n = 4 biological replicates. **c** Naïve B cells isolated from *Mb1*^{Cre} and *Mct1*$^{f/f}$*Mb1*^{Cre} mice were labeled with Ki67 and treated as in (**b**). Ki67 was measured at day 0, 1, 2 and 3 after stimulation, n = 4 biological replicates. Data are presented as mean ± SD, unpaired two-tailed t-test, n.s, not significant.

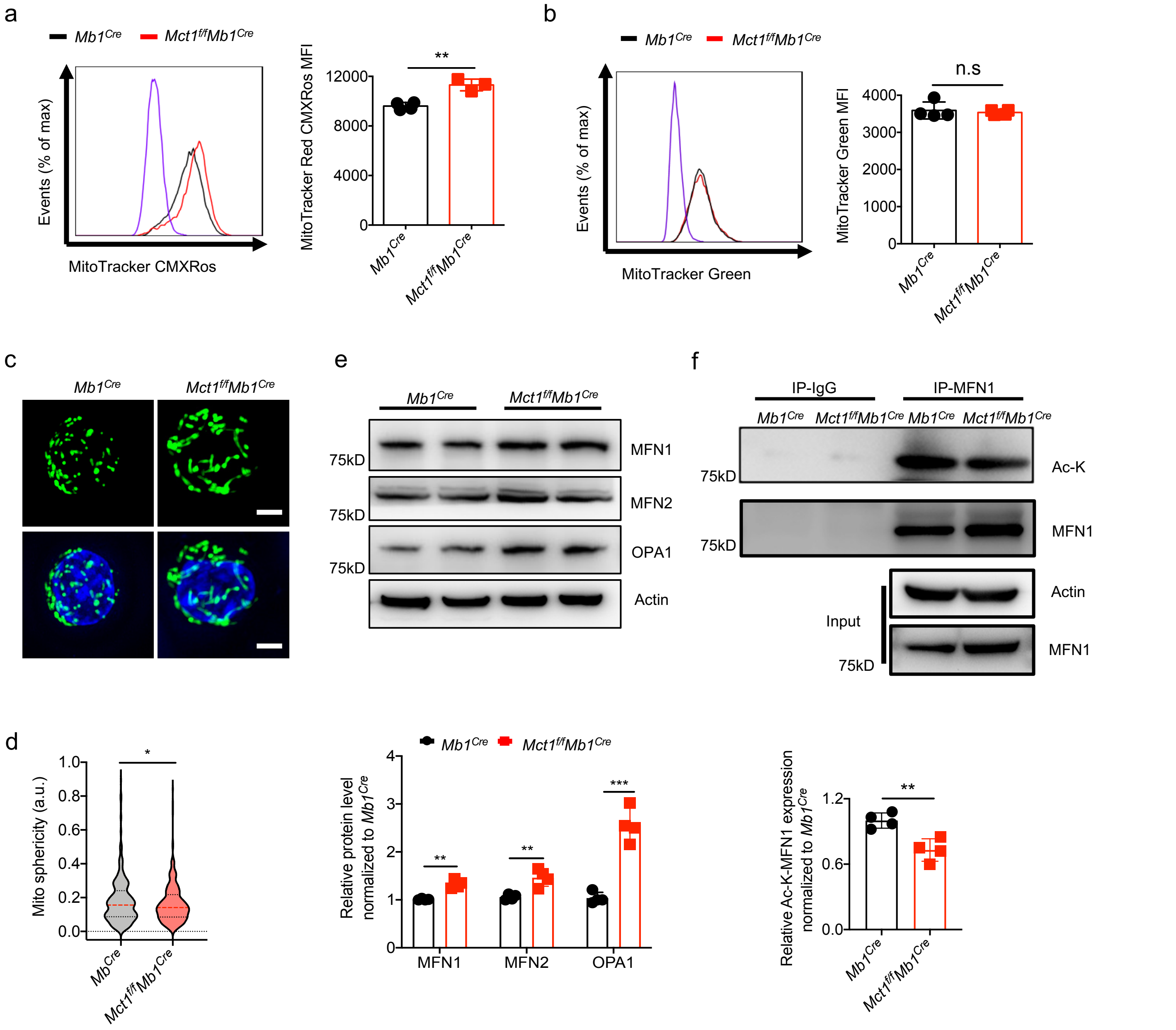

Supplementary Fig.9 *Mct1* deficiency induces a fusion of mitochondria in B cells.
a-b The naïve B cells isolated from *Mb1*^{Cre} and *Mct1*$^{f/f}$*Mb1*^{Cre} mice were stimulated with LPS and IL-4 for 2 days. The cells were stained with Mito-Tracker CMXRos (**a**) and Mito-Tracker Green (**b**) and without staining (purple), then analyzed by flow cytometry. Representative histograms (left), relative MFI (right), *Mb1*^{Cre} (n = 4 biological replicates) and *Mct1*$^{f/f}$*Mb1*^{Cre} (n = 3 biological replicates). **c-d** The mitochondrial morphology in live B cells after LPS and IL-4 activation for 2 days, cultured by using commercialized Hessian-SIM (High intelligent and Sensitive Microscope, HIS-SIM). Mitochondria are green (Mitotracker Deep Red) and nuclei are blue (Hoechst 42). Scale Bar, 2 μm (**c**). Mitochondrial sphericity was determined by Image J. *Mb1*^{Cre}: *n* = 397 mitochondria, *Mct1*$^{f/f}$*Mb1*^{Cre}: *n* = 425 mitochondria (**d**). **e** Western blotting bands and their quantification analysis of MFN1, MFN2 and OPA1 in B cells after LPS/IL-4 activation for 2 days, n = 4 biological replicates. **f** Naive B cells isolated from *Mb1*^{Cre} and *Mct1*$^{f/f}$*Mb1*^{Cre} mice were stimulated with LPS and IL-4 for 3 days. Cells were harvested for immunoprecipitation (IP). Western blotting analysis of MFN1 and acetylation of MFN1 by Ac-K antibody, n = 4 biological replicates. Data are presented as mean ± SD, unpaired two-tailed t test, * $p < 0.05$, ** $p < 0.01$, *** $p < 0.001$, n.s, not significant. Source data are provided as a Source Data file.

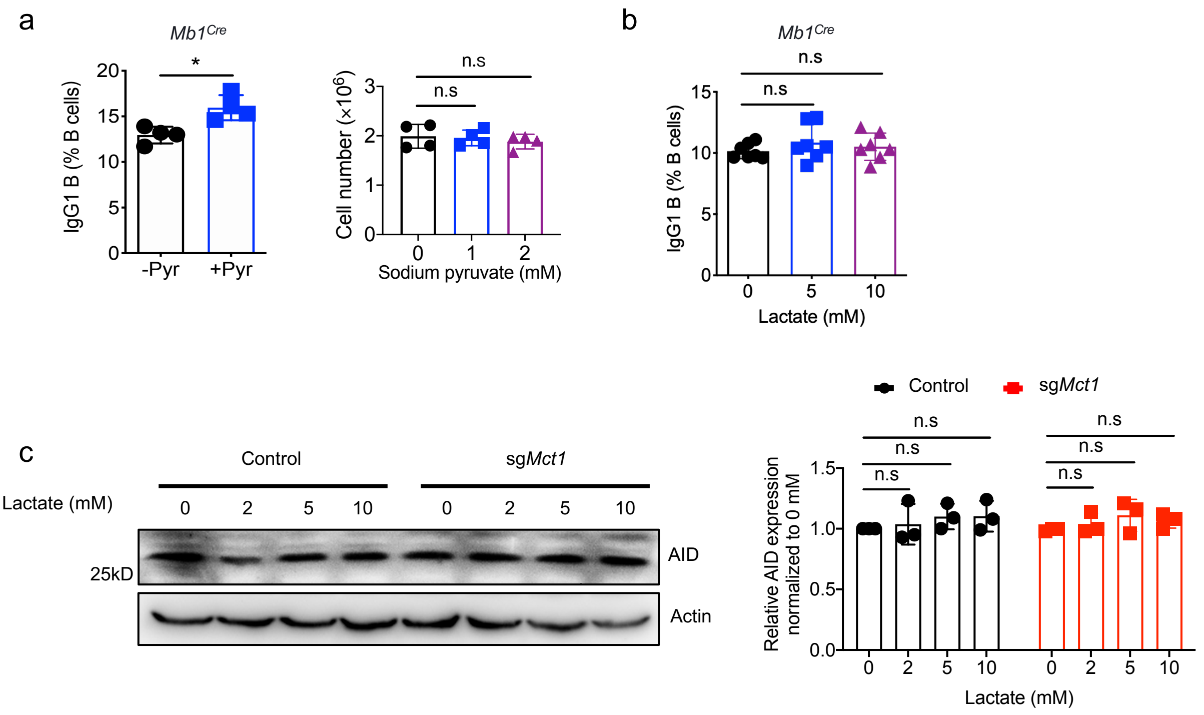

Supplementary Fig.10 The pyruvate is required for the expression of AID. a Naïve B cells isolated from *Mb1*^{Cre} mice were cultured with the different concentration of sodium pyruvate, adding LPS and IL-4 by for 3 days. The cells were collected for cell counting (n = 4 biological replicates). **b** The proportion of IgG1 B cells was analyzed with dose lactate (n = 4 biological replicates) for 3 days by flow cytometry. **c** The expression of AID was detected with dose lactate for 24h in the A20 cells by western blotting, n = 3 biological replicates. Data are presented as mean ± SD, unpaired two-tailed t-test, n.s, not significant. Source data are provided as a Source Data file.

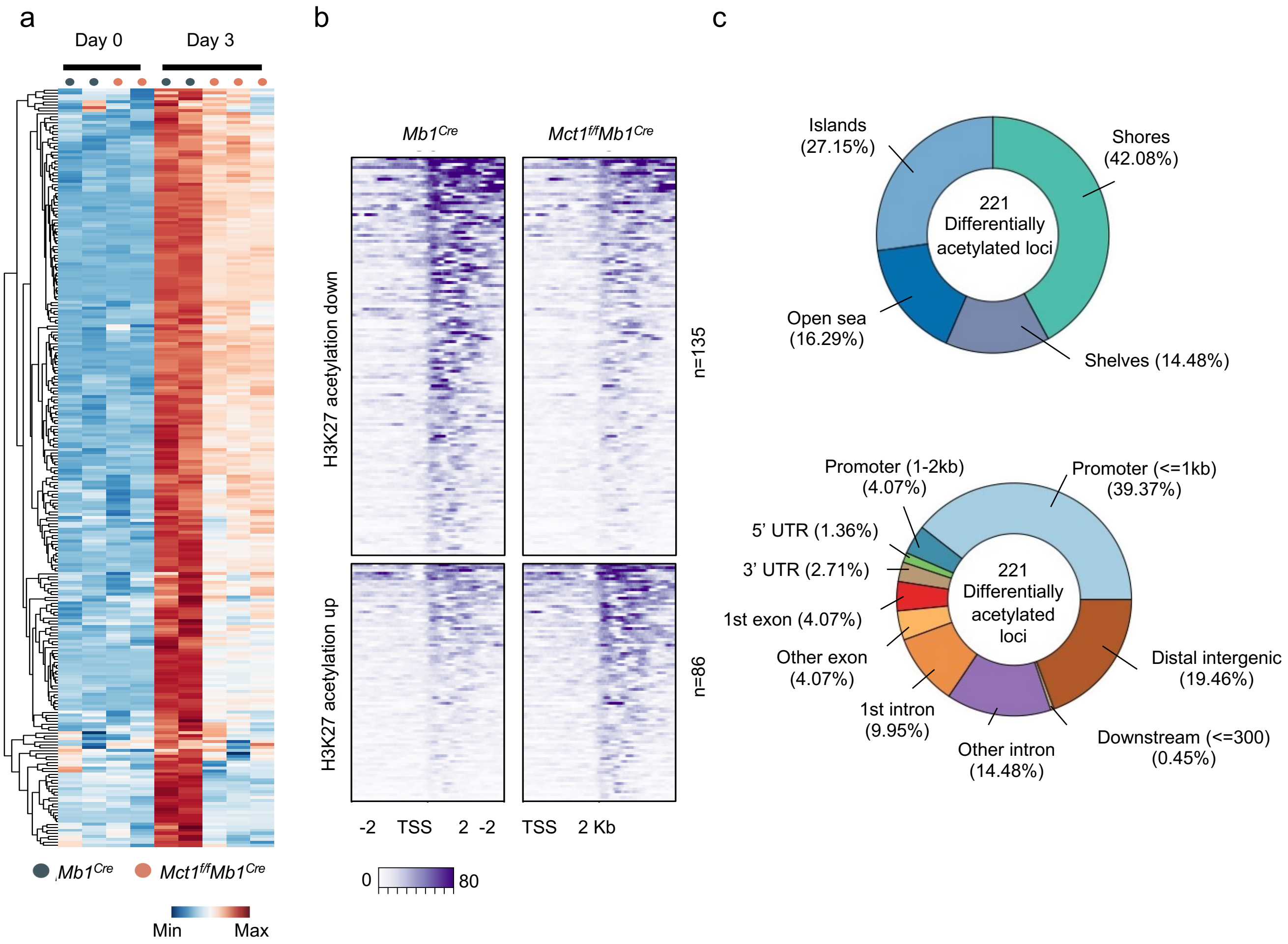

Supplementary Fig.11 *Mct1* deficiency inhibits the acetylation of in B cells. a The RNA-seq heat map of naïve and primary B cells treated with LPS and IL-4 for 3 days. **b** Heatmap of 221 differentially H3K27 acetylated loci in the primary B cells from $Mb1^{Cre}$ and $Mct1^{f/f}Mb1^{Cre}$ mice with treated LPS and IL-4 for 3 days. 85 of H3K27 acetylated loci were increased and 135 of H3K27 acetylated loci were decreased in *Mct1*-deficient B cells compared with WT control B cells. **c** Mapping of 221 differentially acetylated loci to CpG-rich elements (top) and gene bodies (bottom).

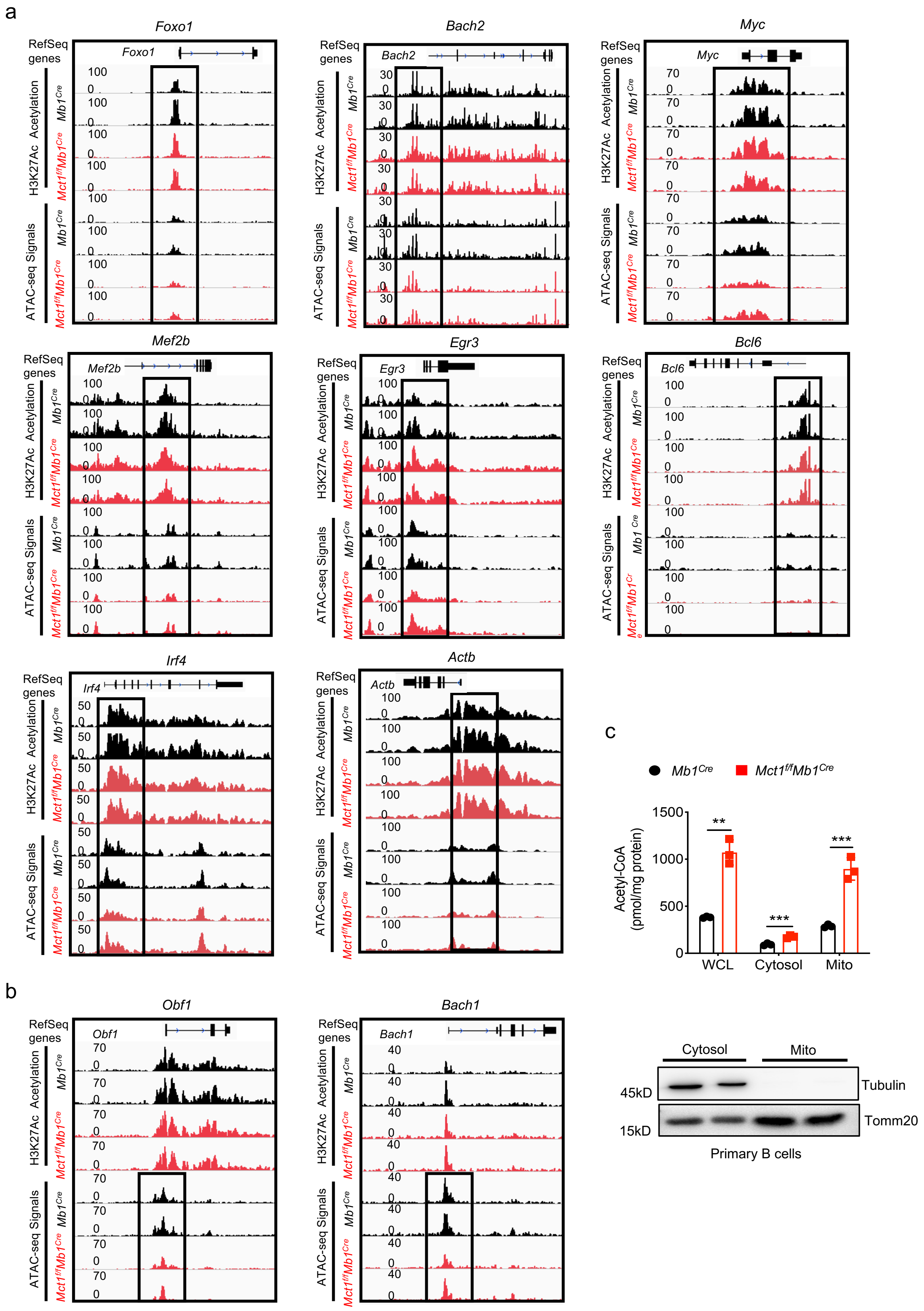

Supplementary Fig.12 Analysis of acetyation in B cells. a Analysis of H3K27 acetylation at the housekeeping gene *Actb*, GC formation genes including *Foxo1, Bach2, Myc, Mef2b, Egr3, Bcl6* and *Irf4* gene promoter with ChIP-seq and ATAC-seq in B cells from $Mb1^{Cre}$ and $Mct1^{f/f}Mb1^{Cre}$ mice stimulated with LPS and IL-4 for 3 days. **b** Analysis of H3K27 acetylation at the *Obf1* and *Bach1* gene promoter with ChIP-seq and ATAC-seq in B cells from $Mb1^{Cre}$ and $Mct1^{f/f}Mb1^{Cre}$ mice stimulated with LPS and IL-4 for 3 days. **c** Acetyl-CoA expression in mitochondria and cytoplasm was measured, n = 3 biological replicates. Data are presented as mean ± SD, unpaired two-tailed t-test, ** $p < 0.01$, *** $p < 0.001$.

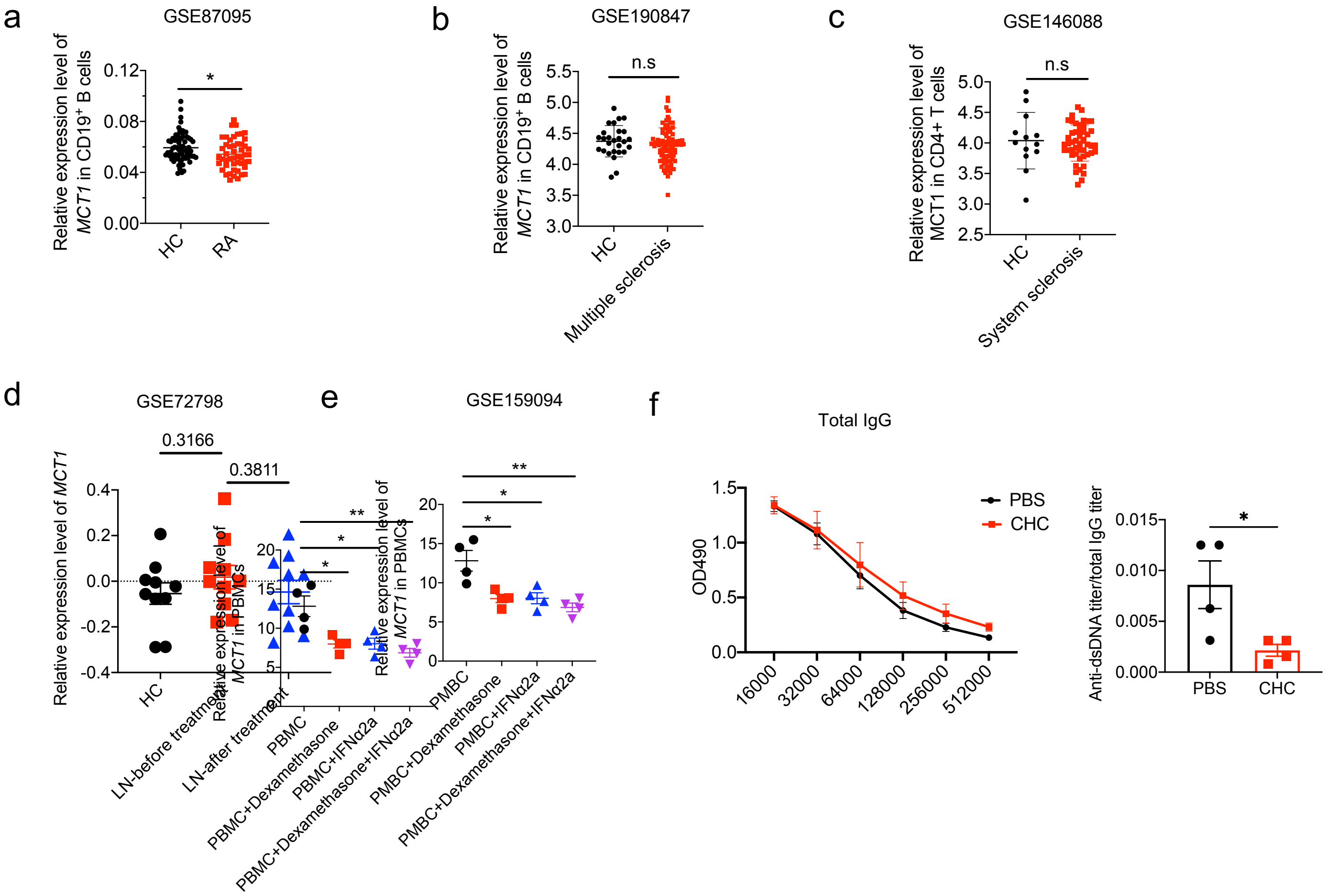

Supplementary Fig.13 The expression of Mct1 in the treatment of SLE. a MCT1 expression in B cells of RA patients and healthy controls, from data of GSE87095. **b** MCT1 expression in B cells of MS patients and healthy controls, from data of GSE190847. **c** MCT1 expression in B cells of SS patients and healthy controls, from data of GSE190847. **d** MCT1 expression in PBMCs upon conventional immunosuppressive drugs treatment of patients with lupus nephritis from data of GSE72798. **e** *MCT1* expression in PBMCs with different types of *in vitro* treatment from data of GSE159094. **f** The total IgG titer or anti-dsDNA antibody titer in WT mice with or without CHC treatment after bm12 cell transfer.

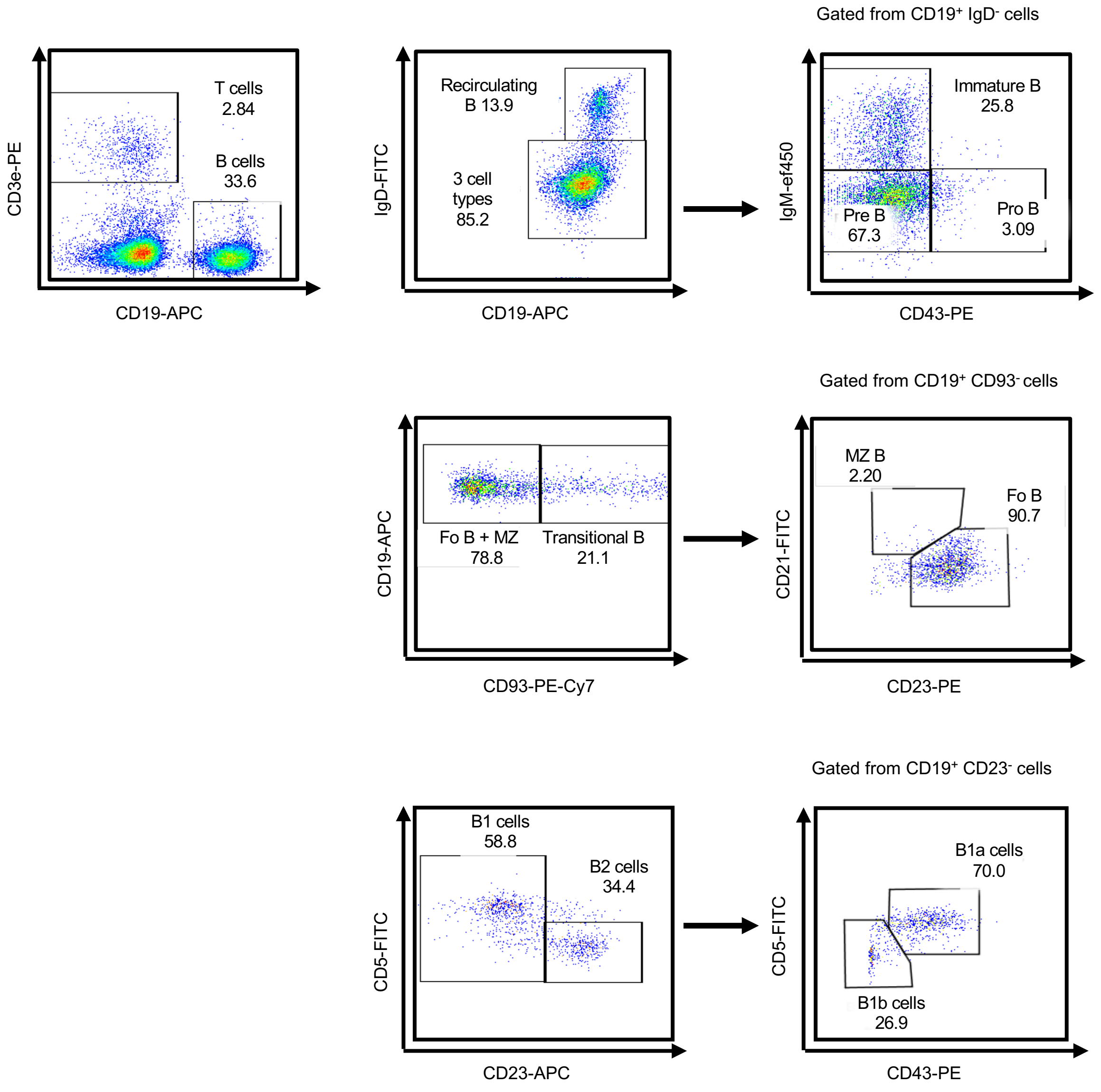

Supplementary Fig.14 Different cell populations of B cells development. Proportion of T cells ($CD19^-CD3e^+$), B cells ($CD19^+CD3e^-$), Recirculating mature B cells ($CD19^+IgD^+$), Immature B cells ($CD19^+IgD^-IgM^+CD43^-$), Pro B cells ($CD19^+IgD^-IgM^-CD43^+$), Pre B cells ($CD19^+IgD^-IgM^-CD43^-$), Transitional B cells ($CD19^+CD93^+$), Follicular B cells ($CD19^+CD93^-CD23^+CD21^{low}$), Marginal B cells ($CD19^+CD93^-CD23^-CD21^{high}$), B1a ($CD19^+CD23^-CD5^+CD43^+$), B1b ($CD19^+CD23^-CD5^-CD43^-$) and B2 cells ($CD19^+CD23^+$) in bone marrow (BM), spleen (SP), peritoneal cavity (PC), or lymph node (LN), analyzed by flow cytometry. Arrows show subsequent gated population.

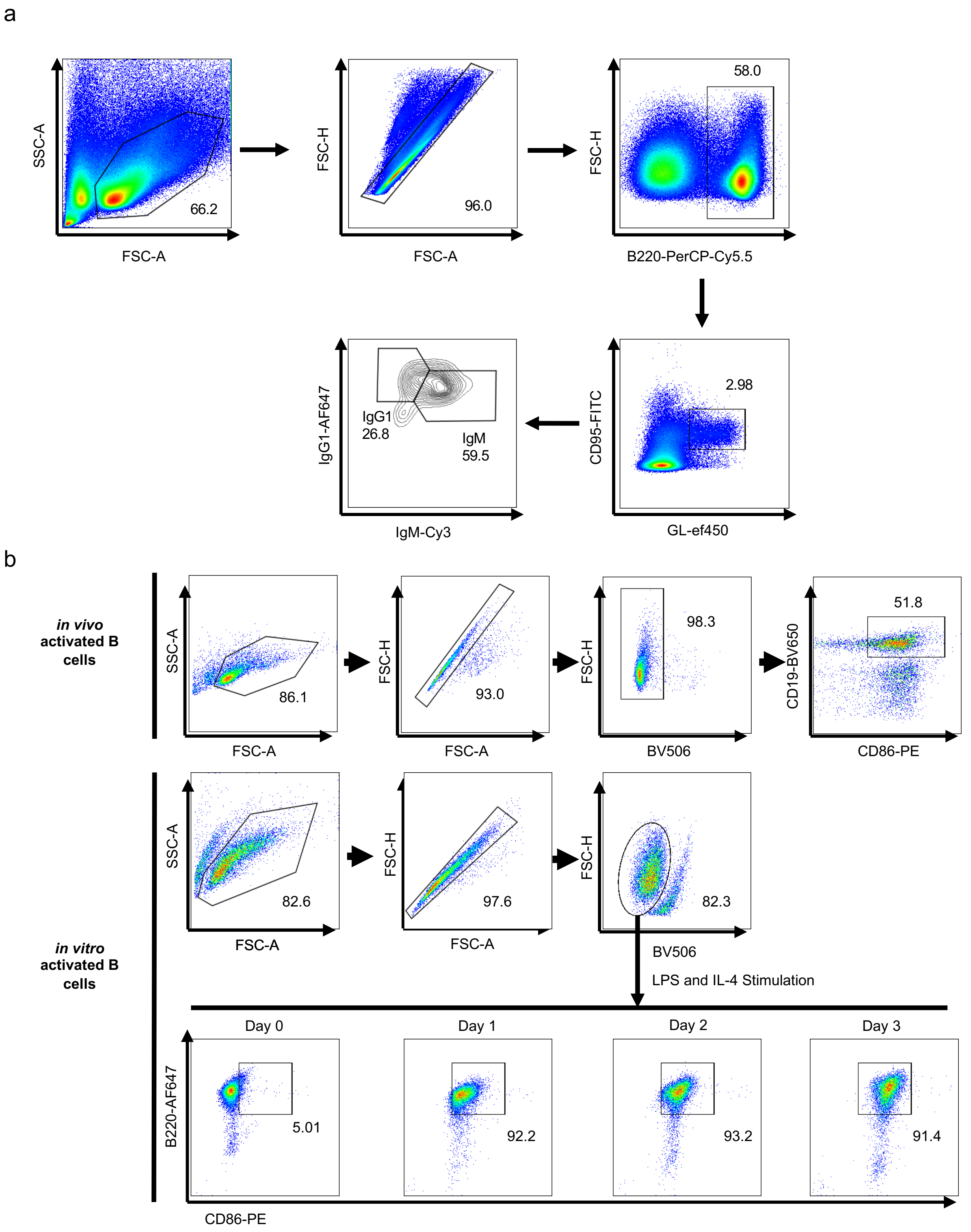

Supplementary Fig.15 Different cell populations of IgG1 B cells and IgM B cells.a Representative FACS plots are shown to determine frequency of indicated target populations - IgG1 GCBC and IgM GCBC. **b** Representative FACS plots are shown to determine frequency of indicated target populations. Our subject, *in vivo*- and *in vitro*-activated B cells, are B220^{+}CD86^{+} after NP-LPS immunization and LPS and IL-4 stimulation respectively.

Supplementary Data

Supplementary Data S1. Primers used for real-time PCR

Supplementary Data S2. Antibody Dilution and Catalogue Number

Supplementary Data S3. RNA-seq of B cells with LPS and IL-4 treatment for 3 days

Supplementary Data S4. 13C-labeled glucose flux analysis of day 0 B cells

Supplementary Data S5. 13C-labeled glucose flux analysis of day 2 B cells

Supplementary Data S6. Metabonomics analysis of day 3 B cells

Supplementary Data S7. Histone proteomics mass spectrometry data

Source Data
