# Supplementary material for: MCT1-governed pyruvate metabolism is essential for antibody class-switch recombination through H3K27 acetylation
Source: Nat Commun. 2024 Jan 2;15:163. doi: 10.1038/s41467-023-44540-0 (PMC10762154; doi:10.1038/s41467-023-44540-0)
Supplement: Supplementary file 3 — Description of Additional Supplementary Files [file 41467_2023_44540_MOESM3_ESM.pdf]

Description of Additional Supplementary Files

File Name: Supplementary Data S1
Description: Primers used for real-time PCR

File Name: Supplementary Data S2
Description: Antibody Dilution and Catalogue Number

File Name: Supplementary Data S3
Description: RNA-seq of B cells with LPS and IL-4 treatment for 3 days

File Name: Supplementary Data S4
Description: 13C-labeled glucose flux analysis of day 0 B cells

File Name: Supplementary Data S5
Description: 13C-labeled glucose flux analysis of day 2 B cells

File Name: Supplementary Data S6
Description: Metabonomics analysis of day 3 B cells

File Name: Supplementary Data S7
Description: Histone proteomics mass spectrometry data
