# Supplementary material for: MCT1-governed pyruvate metabolism is essential for antibody class-switch recombination through H3K27 acetylation
Source: Nat Commun. 2024 Jan 2;15:163. doi: 10.1038/s41467-023-44540-0 (PMC10762154; doi:10.1038/s41467-023-44540-0)
Supplement: Supplementary file 11 — Reporting Summary [file 41467_2023_44540_MOESM11_ESM.pdf]

nature portfolio

Corresponding author(s): Ligong Chen

Last updated by author(s): Nov 22, 2023

Reporting Summary

Nature Portfolio wishes to improve the reproducibility of the work that we publish. This form provides structure for consistency and transparency in reporting. For further information on Nature Portfolio policies, see our Editorial Policies and the Editorial Policy Checklist.

Statistics

For all statistical analyses, confirm that the following items are present in the figure legend, table legend, main text, or Methods section.

n/a	Confirmed	
☐	☒	The exact sample size (*n*) for each experimental group/condition, given as a discrete number and unit of measurement
☐	☒	A statement on whether measurements were taken from distinct samples or whether the same sample was measured repeatedly
☐	☒	The statistical test(s) used AND whether they are one- or two-sided<br>*Only common tests should be described solely by name; describe more complex techniques in the Methods section.*
☐	☒	A description of all covariates tested
☐	☒	A description of any assumptions or corrections, such as tests of normality and adjustment for multiple comparisons
☐	☒	A full description of the statistical parameters including central tendency (e.g. means) or other basic estimates (e.g. regression coefficient) AND variation (e.g. standard deviation) or associated estimates of uncertainty (e.g. confidence intervals)
☐	☒	For null hypothesis testing, the test statistic (e.g. *F*, *t*, *r*) with confidence intervals, effect sizes, degrees of freedom and *P* value noted<br>*Give P values as exact values whenever suitable.*
☒	☐	For Bayesian analysis, information on the choice of priors and Markov chain Monte Carlo settings
☒	☐	For hierarchical and complex designs, identification of the appropriate level for tests and full reporting of outcomes
☒	☐	Estimates of effect sizes (e.g. Cohen's *d*, Pearson's *r*), indicating how they were calculated

Our web collection on statistics for biologists contains articles on many of the points above.

Software and code

Policy information about availability of computer code

Data collection	Seahorse XFe-96 software, HIS-SIM, Eletron microscopy, Confocal microscope (Ziess, LSM780)
Data analysis	Prism 8 software (GraphPad), Image J (v1.53t), Flow Jo_v10,LC-MS/MS

For manuscripts utilizing custom algorithms or software that are central to the research but not yet described in published literature, software must be made available to editors and reviewers. We strongly encourage code deposition in a community repository (e.g. GitHub). See the Nature Portfolio guidelines for submitting code & software for further information.

Data

Policy information about availability of data

All manuscripts must include a data availability statement. This statement should provide the following information, where applicable:

- Accession codes, unique identifiers, or web links for publicly available datasets
- A description of any restrictions on data availability
- For clinical datasets or third party data, please ensure that the statement adheres to our policy

Affymetrix Microarray Data of GSE10325 and GSE4588 and RNA-seq data of GSE118254 were used in this study. The RNA-seq, ATAC-seq, and Chip-seq datasets supporting the current study are not yet deposited in public repositories until publication.

Research involving human participants, their data, or biological material

Policy information about studies with human participants or human data. See also policy information about sex, gender (identity/presentation), and sexual orientation and race, ethnicity and racism.

Reporting on sex and gender	The sex/gender of human participants was determined based on self-reporting. The human participants providing samples for our study were broadly age- and sex-matched. Compared with males, females have a higher incidence of SLE. Due to the small sample size, our study was not sufficiently powered to detect differences according to sex/gender.
Reporting on race, ethnicity, or other socially relevant groupings	All subjects were of Han Chinese origin.
Population characteristics	PBMCs Samples from a total of 24 SLE patients and 19 healthy controls by Peking University Affiliated People's Hospital were analyzed for this study
Recruitment	PBMCs Samples were collected from a total of 24 SLE patients and 19 healthy controls by Peking University Affiliated People's Hospital for this study. HCs were age- and sex-matched individuals without autoimmune, inflammatory or infectious diseases. The protocol of evaluating human MCT1 transcription was approved by ethics committee for biomedical studies. All the volunteers involved in this current study have given their written consent.
Ethics oversight	The experiments were authorized by Peking University Affiliated People's Hospital. The ethics number is 2019PHB234-02.

Note that full information on the approval of the study protocol must also be provided in the manuscript.

Field-specific reporting

Please select the one below that is the best fit for your research. If you are not sure, read the appropriate sections before making your selection.

☒ Life sciences ☐ Behavioural & social sciences ☐ Ecological, evolutionary & environmental sciences

For a reference copy of the document with all sections, see nature.com/documents/nr-reporting-summary-flat.pdf

Life sciences study design

All studies must disclose on these points even when the disclosure is negative.

Sample size	For each assay, at least 6 mice per RNA-seq or ATAC-seq and at least 20 mice for ChIP-seq with three biological replicates for RNA-seq and two for ATAC-seq and ChIP-seq were used. Mouse numbers were chosen based on preliminary experiments showing they could allow sufficient material for these genomic assays. Replicate numbers were chosen following the ENCODE guidelines for these genomic studies.No statistical method was used to predetermine sample size. Required experimental sample sizes were estimated based on previous established protocols in the field. The sample sizes were adequate as the differences between experimental groups were reproducible. All n values are clearly indicated within the figure legends. We included a minimum of 3 biological replicates for each group/treatment/timepoint.
Data exclusions	One sample from the RNA-seq data set was excluded due to low transcript diversity.
Replication	Three biological replicates for RNA-seq and two for ATAC-seq and ChIP-seq.<br>Two different sgRNAs targeting the same region were used for CRISPR.<br>All attempts at replication were successful.<br>All experiments were performed at least three times with similar results. The findings were reliably reproduced.
Randomization	No relevant as we needed to know the identity of each sample prior to their analyses.To further explore the role of MCT1 in mice, we need to perform genetic identification of mice to determine whether mct1 is knocked out in mice before experiment.
Blinding	Not relevant as we needed to know the identity of each sample prior to their analyses.

Reporting for specific materials, systems and methods

We require information from authors about some types of materials, experimental systems and methods used in many studies. Here, indicate whether each material, system or method listed is relevant to your study. If you are not sure if a list item applies to your research, read the appropriate section before selecting a response.

Materials & experimental systems

n/a	Involved in the study	
☐	☒	Antibodies
☐	☒	Eukaryotic cell lines
☒	☐	Palaeontology and archaeology
☐	☒	Animals and other organisms
☒	☐	Clinical data
☒	☐	Dual use research of concern
☒	☐	Plants

Methods

n/a	Involved in the study	
☐	☒	ChIP-seq
☐	☒	Flow cytometry
☒	☐	MRI-based neuroimaging

Antibodies

Antibodies used

Primary antibodies：
anti-phospho-FOXO1 (Thr24) /FOXO3a (Thr32) (#9464),1:1000, Cell Signaling Technology
anti-phospho-AKT (Ser473) (#9271) ,1:1000, Cell Signaling Technology
anti-HK1 (#2024), 1:1000, Cell Signaling Technology
anti-HK2 (#2867),1:1000, Cell Signaling Technology
anti-GAPDH (#5174),1:1000, Cell Signaling Technology
anti-PKM1/2 (#3106) ,1:1000, Cell Signaling Technology
anti-PKM2 (#4053) ,1:1000, Cell Signaling Technology
anti-LDHA (#3582) ,1:1000, Cell Signaling Technology
anti-PDHA (#3205) ,1:1000, Cell Signaling Technology
anti-H3K27Ac (#8173),1:1000, Cell Signaling Technology
anti-H3 (#4499),1:1000, Cell Signaling Technology
Anti-KI67 (#ab15580),1:1000, Abcam
Anti-LDHB (#14824-1-AP),1:1000, Proteintech
anti-ALDOB (#18065-1-AP) ,1:1000, Proteintech
anti-PC (#16588-1-AP) ,1:1000, Proteintech
MFN1 Polyclonal antibody, 1:1000, Proteintech, cat: #13798-1-AP
MFN2 Polyclonal antibody,1000, Proteintech, cat: #12186-1-AP
OPA1 Polyclonal antibody,1:1000, Proteintech, cat: #27733-1-AP
Anti-Tomm20 antibody (ab186735),,1:1000, Abcam, cat: #ab186735
Pan Acetylation Monoclonal antibody,1:1000, Proteintech, cat: #66289-1-Ig
Anti-β-actin (#30102ES40) ,1:1000, Yeasen
Anti-AID (#392500) ,1:1000, Thermo
Anti-MCT1 (#TA321556) ,1:1000, Origene
Cy™3 AffiniPure Fab Fragment Goat Anti-Mouse IgM, µ chain specific (#115-167-020),1:400,
Af450 anti-GL-7 (#48-5902-80),1:400, eBioscience
ef660 anti-GL-7 (#50-5902-82),1:400, eBioscience
Alexa Fluor 488 anti-CXCR4 (#53-9991-80) ,1:400,eBioscience
ef450 anti-IgM (#48-5790-82),1:400, eBioscience
PerCP/Cyanine5.5 anti-B220 (#103209) ,1:400,Biolegend
FITC anti-CD95 (#152605) ,1:400,Biolegend
PE anti-CD95 (#152607) ,1:400,Biolegend
APC anti-CD86 (#105012),1:400, Biolegend
E anti-CD3 (#100205) ,1:400,Biolegend
APC anti-CD19 (#115511),1:400, Biolegend
FITC anti-IgD (#405703) ,1:400,Biolegend
PE anti-CD43 (#143205),1:400, Biolegend
PE/Cyanine7 anti-CD93 (#136505) ,1:400,Biolegend
PE anti-CD23 (#101607) ,1:400,Biolegend
FITC anti-CD21 (#123407),1:400, Biolegend
FITC anti-CD5 (#100605) ,1:400,Biolegend
Biotin anti-CD19 (#553784) ,1:200,BD Biosciences
anti-CD16/CD32 (#553140) ,1:40,BD Biosciences
Anti-κchain (#1050-01),1:8000, SouthernBiotech
anti-IgM-HRP (#1020-05),1:8000, SouthernBiotech
anti-IgG-HRP (#1030-05) ,1:8000, SouthernBiotech
anti-IgG1-HRP (#1070-05) ,1:8000, SouthernBiotech
anti-IgG2b-HRP (#1090-05) ,1:8000, SouthernBiotech
anti-IgG2c-HRP (#1079-05) ,1:8000, SouthernBiotech
anti-IgG3-HRP (#1100-05) ,1:8000, SouthernBiotech
Alexa Fluor 488 Fab anti-IgM µ chain (#115-547-020) Jackson ImmunoResearch Laboratories
Alexa Fluor 647 Fab anti-IgG1 Fcγ (#115-607-185) Jackson ImmunoResearch Laboratories

Validation

All the antibodies were commercially available and their validation statements are available on the manufacturers' websites.
anti-phospho-FOXO1 (Thr24) /FOXO3a (Thr32) Cell Signaling Technology
Validation information found at:
anti-phospho-FOXO1 (Thr24) /FOXO3a (Thr32) (#9464) Cell Signaling Technology cat: #9464, cation: Nat Commun. 2023 Jul 13;14(1):4162. doi: 10.1038/s41467-023-39715-8, and the antibody was validated (i.e assay and species) by the company using HT29

cells for western blot assay, please refer to the manufacturer's description: https://www.cellsignal.cn/products/primary-antibodies/phospho-foxo1-thr24-foxo3a-thr32-antibody/9464

anti-phospho-AKT (Ser473) (#9271) Cell Signaling Technology
Validation information found at:
Phospho-Akt (Ser473) Antibody, Cell Signaling Technology, cat: #9271, cation: J Clin Invest. 2023 Oct 2;133(19): e169173. doi: 10.1172/JCI169173, and the antibody was validated (i.e assay and species) by the company using NIH/3T3 cells for western blot assay, please refer to the manufacturer's description: https://www.cellsignal.cn/products/primary-antibodies/phospho-akt-ser473-d9e-xp-rabbit-mab/4060

anti-HK1 (#2024), Cell Signaling Technology
Validation information found at:
Hexokinase I (C35C4) Rabbit mAb, Cell Signaling Technology, cat: #2024, cation: Cell Death Discov. 2023 Oct 2;9(1):364. doi: 10.1038/s41420-023-01648-y, and the antibody was validated (i.e assay and species) by the company using MCF-7, U87MG, 293, U118MG, NIH/3T3, C2C12 and nIMCD3 cells for western blot assay, please refer to the manufacturer's description:
https://www.cellsignal.cn/products/primary-antibodies/hexokinase-i-c35c4-rabbit-mab/2024

anti-HK2 (#2867) Cell Signaling Technology
Validation information found at:
Hexokinase II (C64G5) Rabbit mAb, Cell Signaling Technology, cat: #2867, cation: Cells. 2023 Aug 10;12(16):2043. doi: 10.3390/cells12162043, and the antibody was validated (i.e assay and species) by the company using A172, Hela, COS-7 and RD cells for western blot assay, please refer to the manufacturer's description: https://www.cellsignal.cn/products/primary-antibodies/hexokinase-ii-c64g5-rabbit-mab/2867

anti-GAPDH (#5174) Cell Signaling Technology
Validation information found at:
GAPDH (D16H11) XP® Rabbit mAb, Cell Signaling Technology, cat: #5174, cation: Genes Dis. 2023 Mar 24;11(2):964-977. doi: 10.1016/j.gendis.2023.02.012. eCollection 2024 Mar, and the antibody was validated (i.e assay and species) by the company using C6, Hela, COS-7 and NIH/3T3 cells for western blot assay, please refer to the manufacturer's description:
https://www.cellsignal.cn/products/primary-antibodies/gapdh-d16h11-xp-rabbit-mab/5174

anti-PKM1/2 (#3106) Cell Signaling Technology
Validation information found at:
PKM1/2 (C5E6) Rabbit mAb, Cell Signaling Technology, cat: #3106, cation: Cell Metab. 2020 Sep 1;32(3):420-436.e12. doi: 10.1016/j.cmet.2020.07.008. Epub 2020 Aug 7, and the antibody was validated (i.e assay and species) by the company using MCF-7, RD, A204 and C2C12 cells for western blot assay, please refer to the manufacturer's description:
https://www.cellsignal.cn/products/primary-antibodies/pkm1-2-c5e6-rabbit-mab/3106

anti-PKM2 (#4053) Cell Signaling Technology
Validation information found at:
PKM2 (D78A4) XP® Rabbit mAb, Cell Signaling Technology, cat: #4053, cation: iScience. 2023 Sep 9;26(10):107869. doi: 10.1016/j.isci.2023.107869. eCollection 2023 Oct 20, and the antibody was validated (i.e assay and species) by the company using Hela, C6, A204, NIH/3T3 cells and mouse skeletal muscle for western blot assay, please refer to the manufacturer's description:
https://www.cellsignal.cn/products/primary-antibodies/pkm2-d78a4-xp-rabbit-mab/4053

anti-LDHA (#3582) Cell Signaling Technology
Validation information found at:
LDHA (C4B5) Rabbit mAb, Cell Signaling Technology, cat: #3582, cation: Nat Commun
. 2023 Sep 22;14(1):5894. doi: 10.1038/s41467-023-41484-3, and the antibody was validated (i.e assay and species) by the company using Hela, RD, MCF-7 and COS-7 cells for western blot assay, please refer to the manufacturer's description:
https://www.cellsignal.cn/products/primary-antibodies/ldha-c4b5-rabbit-mab/3582

anti-PDHA (#3205) Cell Signaling Technology
Validation information found at:
Pyruvate Dehydrogenase (C54G1) Rabbit mAb, Cell Signaling Technology, cat: #3205, cation: Nat Metab. 2023 Aug;5(8):1290-1302. doi: 10.1038/s42255-023-00835-6. Epub 2023 Jul 17, and the antibody was validated (i.e assay and species) by the company using HepG2, 293, Hela and A204 cells for western blot assay, please refer to the manufacturer's description: https://www.cellsignal.cn/products/primary-antibodies/pyruvate-dehydrogenase-c54g1-rabbit-mab/3205

anti-H3K27Ac (#8173) Cell Signaling Technology
Validation information found at:
Acetyl-Histone H3 (Lys27) (D5E4) XP® Rabbit mAb, Cell Signaling Technology, cat: #8173, cation: Nat Commun. 2023 Sep 22;14(1):5916. doi: 10.1038/s41467-023-41585-z, and the antibody was validated (i.e assay and species) by the company using Hela and C2C12 cells for western blot assay, please refer to the manufacturer's description: https://www.cellsignal.cn/products/primary-antibodies/acetyl-histone-h3-lys27-d5e4-xp-rabbit-mab/8173

anti-H3 (#4499) Cell Signaling Technology
Validation information found at:
Histone H3 (D1H2) XP® Rabbit mAb, Cell Signaling Technology, cat: #4499, cation: Nat Commun. 2023 Sep 22;14(1):5916. doi: 10.1038/s41467-023-41585-z, and the antibody was validated (i.e assay and species) by the company using Hela, NIH/3T3, C6 and COS cells for western blot assay, please refer to the manufacturer's description: https://www.cellsignal.cn/products/primary-antibodies/histone-h3-d1h2-xp-rabbit-mab/4499

Anti-KI67 (#ab15580) Abcam
Validation information found at:
Anti-Ki67 antibody (ab15580), Abcam, cat: # ab15580, cation: Stem Cell Rev Rep. 2023 Jan;19(1):133-154. doi: 10.1007/s12015-021-10221-y. Epub 2021 Aug 22, and the antibody was validated (i.e assay and species) by the company using Ki67 knockout HAP1 cells, please refer to the manufacturer's description: https://www.abcam.cn/ki67-antibody-ab15580.html

Anti-LDHB (#14824-1-AP) Proteintech
Validation information found at:
LDHB Polyclonal antibody, Proteintech , cat: #14824-1-AP, cation: Cell Death Differ. 2021 Sep;28(9):2673-2689. doi: 10.1038/s41418-021-00777-0. Epub 2021 May 26, and the antibody was validated (i.e assay and species) by the company using LDHB knockdown Hela cells, please refer to the manufacturer's description:
https://www.ptgcn.com/products/LDHB-Antibody-14824-1-AP.htm

anti-ALDOB (#18065-1-AP) Proteintech
Validation information found at:
ALDOB Polyclonal antibody, Proteintech , cat: #18065-1-AP, cation: Nature. 2017 Aug 3;548(7665):112-116. doi: 10.1038/nature23275. Epub 2017 Jul 19. and the antibody was validated (i.e assay and species) by the company using mouse monkey, mouse skeletal muscle and rat kidney, please refer to the manufacturer's description:
https://www.ptgcn.com/products/ALDOB-Antibody-18065-1-AP.htm

anti-PC (#16588-1-AP) Proteintech
Validation information found at:
Pyruvate Carboxylase Polyclonal antibody, Proteintech, cat: #16588-1-AP, cation: Cell Metab. 2023 Oct 3;35(10):1830-1843.e5. doi: 10.1016/j.cmet.2023.07.013, and the antibody was validated (i.e assay and species) by the company using PC knockdown HepG2 cells, please refer to the manufacturer's description:
https://www.ptgcn.com/products/PC-Antibody-16588-1-AP.htm

MFN1 Polyclonal antibody, Proteintech, cat: #13798-1-AP, cation: Cell. 2021 May 27;184(11):2896-2910.e13. doi: 10.1016/j.cell.2021.04.027, and the antibody was validated (i.e assay and species) by the company using HSC-T6, T-47D cells, mouse brain, mouse kidney and mouse liver, please refer to the manufacturer's description:
https://www.ptgcn.com/products/MFN1-Antibody-13798-1-AP.htm#publications

MFN2 Polyclonal antibody, Proteintech, cat: #12186-1-AP, cation: Cell. 2021 May 27;184(11):2896-2910.e13. doi: 10.1016/j.cell.2021.04.027, and the antibody was validated (i.e assay and species) by the company using mouse brain, mouse liver, rat brain and rat heart, please refer to the manufacturer's description:
https://www.ptgcn.com/products/MFN2-Antibody-12186-1-AP.htm#publications

OPA1 Polyclonal antibody, Proteintech, cat: #27733-1-AP, cation: Mol Cell. 2022 May 19;82(10):1821-1835.e6. doi: 10.1016/j.molcel.2022.03.016. Epub 2022 Apr 4, and the antibody was validated (i.e assay and species) by the company using A431, Hela, HepG2 cells, mouse brain and rat brain, please refer to the manufacturer's description:
https://www.ptgcn.com/products/OPA1-Antibody-27733-1-AP.htm

Anti-Tomm20 antibody (ab186735), Abcam, cat: #ab186735, cation: Nat Commun. 2022 Feb 28;13(1):1071. doi: 10.1038/s41467-022-28677-y, and the antibody was validated (i.e assay and species) by the company using Hela, HepG2 and SH-SY5Y cells, please refer to the manufacturer's description:

Pan Acetylation Monoclonal antibody, Proteintech, cat: #66289-1-Ig, cation: Neuron. 2021 Mar 17;109(6):957-970.e8. doi: 10.1016/j.neuron.2021.01.005. Epub 2021 Jan 26, and the antibody was validated (i.e assay and species) by the company using Hela cells, please refer to the manufacturer's description: https://www.ptgcn.com/products/Pan-Acetylation-Antibody-66289-1-Ig.htm#publications

Anti-β-actin (#30102ES40) Yeasen
Validation information found at:
Anti-β-actin antibody, Yeasen, cat: #30102ES40, cation: Front Immunol. 2021 May 20:12:626493. doi: 10.3389/fimmu.2021.626493. eCollection 2021, and the antibody was validated (i.e assay and species) by the company using Hela, human fetal kidney, NIH/3T3 and PC12 cells, please refer to the manufacturer's description:
https://www.yeasen.com/products/detail/879

Anti-AID (#392500) Thermo
Validation information found at:
AID Monoclonal Antibody (ZA001), Thermo, cat: #392500, cation: J Immunol. 2015 Apr 1;194(7):3065-78. doi: 10.4049/jimmunol.1401896. Epub 2015 Mar 4, and the antibody was validated (i.e assay and species) by the company using germinal center B cells, please refer to the manufacturer's description:
https://www.thermofisher.cn/cn/zh/antibody/product/AID-Antibody-clone-ZA001-Monoclonal/39-2500

Anti-MCT1 (#TA321556) Origene
Validation information found at:
Monocarboxylic acid transporter 1 (SLC16A1) Rabbit Polyclonal Antibody, Origene, cat: #TA321556, cation: Cell Stem Cell. 2019 Dec 5;25(6):754-767.e9. doi: 10.1016/j.stem.2019.09.009. Epub 2019 Nov 21, and the antibody was validated (i.e assay and species) by the company using human liver cancer, please refer to the manufacturer's description:
https://www.origene.com.cn/catalog/antibodies/primary-antibodies/ta321556/monocarboxylic-acid-transporter-1-slc16a1-rabbit-polyclonal-antibody

Cy™3 AffiniPure Fab Fragment Goat Anti-Mouse IgM, µ chain specific (#115-167-020)

Validation information found at:
Cy™3 AffiniPure Fab Fragment Goat Anti-Mouse IgM, µ chain specific Antibody, Jackson ImmunoResearch Laboratories, cat: #115-167-020, cation: Elife. 2019 Jun 3:8:e44574. doi: 10.7554/eLife.44574, and the antibody was validated (i.e assay and species) by the company, please refer to the manufacturer's description:
https://www.jacksonimmuno.com/catalog/products/115-167-020

Alexa Fluor 488 Fab anti-IgM µ chain (#115-547-020) Jackson ImmunoResearch Laboratories
Validation information found at:
Alexa Fluor® 488 AffiniPure Fab Fragment Goat Anti-Mouse IgM, µ chain specific Antibody, Jackson ImmunoResearch Laboratories, cat: #115-547-020, cation: Nat Commun. 2018 Aug 17;9(1):3288. doi: 10.1038/s41467-018-05771-8, and the antibody was validated (i.e assay and species) by the company, please refer to the manufacturer's description:
https://www.jacksonimmuno.com/catalog/products/115-547-020

Alexa Fluor 647 Fab anti-IgG1 Fcγ (#115-607-185) Jackson ImmunoResearch Laboratories
Validation information found at:
Alexa Fluor® 647 AffiniPure Fab Fragment Goat Anti-Mouse IgG1, Fcγ fragment specific Antibody, Jackson ImmunoResearch Laboratories, cat: #115-607-185, cation: Nat Commun. 2020 Apr 27;11(1):1982. doi: 10.1038/s41467-020-15906-5, and the antibody was validated (i.e assay and species) by the company, please refer to the manufacturer's description:
https://www.jacksonimmuno.com/catalog/products/115-607-185

Af450 anti-GL-7 (#48-5902-80) eBioscience
Validation information found at:
GL7 Monoclonal Antibody (GL-7 (GL7)), eFluor™ 450, eBioscience™, cat: #48-5902-80, cation: iScience. 2022 Apr 15;25(4):104043. doi: 10.1016/j.isci.2022.104043. Epub 2022 Mar 11, and the antibody was validated (i.e assay and species) by the company using C57BL/6 splenocytes, please refer to the manufacturer's description:
https://www.thermofisher.cn/cn/zh/antibody/product/GL7-Antibody-clone-GL-7-GL7-Monoclonal/48-5902-80

ef660 anti-GL-7 (#50-5902-82) eBioscience
Validation information found at:
GL7 Monoclonal Antibody (GL-7 (GL7)), eFluor™ 660, eBioscience™, cat: #50-5902-82, cation: Cell. 2023 Jan 5;186(1):147-161.e15. doi: 10.1016/j.cell.2022.11.032. Epub 2022 Dec 23, and the antibody was validated (i.e assay and species) by the company using C57BL/6 splenocytes, please refer to the manufacturer's description:
https://www.thermofisher.cn/cn/zh/antibody/product/GL7-Antibody-clone-GL-7-GL7-Monoclonal/50-5902-82

Alexa Fluor 488 anti-CXCR4 (#53-9991-80) eBioscience
Validation information found at:
CD184 (CXCR4) Monoclonal Antibody (2B11), Alexa Fluor™ 488, eBioscience™, cat: #53-9991-80, cation: Nat Immunol. 2021 Feb;22(2):240-253. doi: 10.1038/s41590-020-00827-8. Epub 2021 Jan 11, and the antibody was validated (i.e assay and species) by the company using C57BL/6 thymocytes, please refer to the manufacturer's description:
https://www.thermofisher.cn/cn/zh/antibody/product/CD184-CXCR4-Antibody-clone-2B11-Monoclonal/53-9991-80

ef450 anti-IgM (#48-5790-82) eBioscience
Validation information found at:
IgM Monoclonal Antibody (II/41), eFluor™ 450, eBioscience™, cat: #48-5790-82, cation: Nature. 2021 Jul;595(7866):278-282. doi: 10.1038/s41586-021-03676-z. Epub 2021 Jun 7. Epub 2021 Jan 11, and the antibody was validated (i.e assay and species) by the company using C57BL/6 bone marrow cells, please refer to the manufacturer's description:
https://www.thermofisher.cn/cn/zh/antibody/product/IgM-Antibody-clone-II-41-Monoclonal/48-5790-82

PerCP/Cyanine5.5 anti-B220 (#103209) Biolegend
Validation information found at:
PE/Cyanine5 anti-mouse/human CD45R/B220 Antibody, Biolegend, cat: #103209, cation: Cancer Cell. 2020 May 11;37(5):690-704.e8. doi: 10.1016/j.ccell.2020.03.022. Epub 2020 Apr 23, and the antibody was validated (i.e assay and species) by the company using C57BL/6 mouse splenocytes, please refer to the manufacturer's description: https://www.biolegend.com/en-us/products/pe-cyanine5-anti-mouse-human-cd45r-b220-antibody-448

FITC anti-CD95 (#152605) Biolegend
Validation information found at:
FITC anti-mouse CD95 (Fas) Antibody, Biolegend, cat: #152605, cation: Immunity. 2022 Nov 8;55(11):2059-2073.e8. doi: 10.1016/j.immuni.2022.09.014. Epub 2022 Oct 19, and the antibody was validated (i.e assay and species) by the company using C57BL/6 thymocytes, please refer to the manufacturer's description: https://www.biolegend.com/en-us/products/fitc-anti-mouse-cd95-fas-antibody-13897

PE anti-CD95 (#152607) Biolegend
Validation information found at:
PE anti-mouse CD95 (Fas) Antibody, Biolegend, cat: #152607, cation: Immunity. 2018 Dec 18;49(6):1034-1048.e8. doi: 10.1016/j.immuni.2018.10.012, and the antibody was validated (i.e assay and species) by the company using C57BL/6 thymocytes, please refer to the manufacturer's description: https://www.biolegend.com/en-us/products/pe-anti-mouse-cd95-fas-antibody-13907

APC anti-CD86 (#105012) Biolegend
Validation information found at:
APC anti-mouse CD86 Antibody, Biolegend, cat: #105012, cation: Immunity. 2019 Feb 19;50(2):418-431.e6. doi: 10.1016/j.immuni.2019.01.014. Epub 2019 Feb 12, and the antibody was validated (i.e assay and species) by the company using LPS-stimulated (3 days) C57BL/6 mouse splenocytes, please refer to the manufacturer's description: https://www.biolegend.com/en-us/products/apc-anti-mouse-cd86-antibody-2896

PE anti-CD3 (#100205) Biolegend
Validation information found at:
PE anti-mouse CD3 Antibody, Biolegend, cat: #100205, cation: Nat Immunol. 2022 Apr;23(4):605-618. doi: 10.1038/s41590-022-01165-7. Epub 2022 Mar 28, and the antibody was validated (i.e assay and species) by the company using C57BL/6 mouse splenocytes, please refer to the manufacturer's description: https://www.biolegend.com/en-us/products/pe-anti-mouse-cd3-antibody-47

APC anti-CD19 (#115511) Biolegend
Validation information found at:
APC anti-mouse CD19 Antibody, Biolegend, cat: #115511, cation: Immunity. 2017 Aug 15;47(2):268-283.e9. doi: 10.1016/j.immuni.2017.07.008. Epub 2017 Aug 1, and the antibody was validated (i.e assay and species) by the company using C57BL/6 mouse splenocytes, please refer to the manufacturer's description: https://www.biolegend.com/en-us/products/apc-anti-mouse-cd19-antibody-1526

FITC anti-IgD (#405703) Biolegend
Validation information found at:
FITC anti-mouse IgD Antibody, Biolegend, cat: #405703, cation: Nat Commun. 2022 Feb 21;13(1):980. doi: 10.1038/s41467-022-28576-2, and the antibody was validated (i.e assay and species) by the company using C57BL/6 mouse splenocytes, please refer to the manufacturer's description: https://www.biolegend.com/en-us/products/fitc-anti-mouse-igd-1378

PE anti-CD43 (#143205) Biolegend
Validation information found at:
PE anti-mouse CD43 Antibody, Biolegend, cat: #143205, cation: Nat Commun. 2021 Jan 22;12(1):525. doi: 10.1038/s41467-020-20874-x, and the antibody was validated (i.e assay and species) by the company using C57BL/6 mouse splenocytes, please refer to the manufacturer's description: https://www.biolegend.com/en-us/products/pe-anti-mouse-cd43-antibody-7704

PE/Cyanine7 anti-CD93 (#136505) Biolegend
Validation information found at:
PE/Cyanine7 anti-mouse CD93 (AA4.1, early B lineage) Antibody, Biolegend, cat: #136505, cation: Nat Commun. 2021 Jan 22;12(1):525. doi: 10.1038/s41467-020-20874-x, and the antibody was validated (i.e assay and species) by the company using C57BL/6 bone marrow cells, please refer to the manufacturer's description: https://www.biolegend.com/en-us/products/pe-cyanine7-anti-mouse-cd93-aa4-1-early-b-lineage-antibody-6420

Eukaryotic cell lines

Policy information about cell lines and Sex and Gender in Research

Cell line source(s)	A20, were purchased from ATCC (Virginia, United States of America)
Authentication	None of the cell lines used were authenticated outside of validating the indicated genetic changes and confirming the resulting alterations in cellular processes.
Mycoplasma contamination	Cell lines were tested for mycoplasma contamination annually and confirmed to be negative.
Commonly misidentified lines (See ICLAC register)	No commonly misidentified cell lines were used in this study.

Animals and other research organisms

Policy information about studies involving animals; ARRIVE guidelines recommended for reporting animal research, and Sex and Gender in Research

Laboratory animals	C57BL/6J (JAX664) and bm12 (JAX1162) were purchased from the Jackson laboratory. Mb1Cre mice were obtained from the Jackson Laboratory (stock NO.020505). C57BL/6J (B6) background Mct1f/f mice were designed with the CRISPR-Cas9 by the NIBS25 (National Institute of Biological Sciences, Beijing). Two floxed sites were inserted flanking Mct1 exon 1 and intron 3, respectively.Offsprings carrying Mb1-Cre and two copies of the floxed Mct1 allele were used in the experiments as homozygous mutant Mct1f/fMb1Cre mice, and Mb1Cre mice as control, respectively. Mice(male and female, 6-8 weeks) were maintained in separately ventilated cages in a specific pathogen-free (SPF) facility, in a room with temperature and 12/12-h light-dark cycle, and the animals had unlimited access to food and water.
Wild animals	The study did not include wild animals.
Reporting on sex	Both male and female mice were used in the study.
Field-collected samples	The study did not include filed-collected sample.
Ethics oversight	All animal procedures and experiments were carried out in accordance with the guidelines of the Laboratory Animal Research Center of Tsinghua University. The laboratory animal facility has been licensed by the Science and Technology Commission of Beijing Municipality (SYXK-2014-0024) and accredited by Association for Assessment and Accreditation of Laboratory Animal Care International.

Note that full information on the approval of the study protocol must also be provided in the manuscript.

Plants

Seed stocks	N/A
Novel plant genotypes	N/A
Authentication	N/A

ChIP-seq

Data deposition

☒ Confirm that both raw and final processed data have been deposited in a public database such as GEO.

☐ Confirm that you have deposited or provided access to graph files (e.g. BED files) for the called peaks.

Data access links<br>*May remain private before publication.*	ChIP-seq raw data has been deposited in the Gene Expression Omnibus (GEO) under accession number GSE247201 (https://www.ncbi.nlm.nih.gov/geo/query/acc.cgi?acc=GSE247201).
Files in database submission	B cells,H3K27Ac_CHIP,Con-1<br>B cells,H3K27Ac_CHIP,Con-2<br>B cells,H3K27Ac_CHIP,Con-3<br>B cells,H3K27Ac_CHIP,KO-1<br>B cells,H3K27Ac_CHIP,KO-2<br>B cells,H3K27Ac_CHIP,KO-3
Genome browser session<br>(e.g. UCSC)	No longer applicable.

Methodology

Replicates	Three replicates.
Sequencing depth	3.3-5.2 million total reads.Single read 150bp.
Antibodies	anti-H3K27Ac （#8173） Cell Signaling Technology
Peak calling parameters	-f BAM –mfold 0 50 -g mm –nomodel –extsize228 –keep-dup all –qvalue 0.001
Data quality	N/A
Software	Sequencing reads were mapped to the genome using bowtie (version noted above) allowing no mismatch per readalignment and only uniquely aligned reads (-v 1 -m 1). Peaks were called against input using MACS2.

Flow Cytometry

Plots

Confirm that:

☒ The axis labels state the marker and fluorochrome used (e.g. CD4-FITC).

☒ The axis scales are clearly visible. Include numbers along axes only for bottom left plot of group (a 'group' is an analysis of identical markers).

☒ All plots are contour plots with outliers or pseudocolor plots.

☒ A numerical value for number of cells or percentage (with statistics) is provided.

Methodology

Sample preparation	Naïve B cells or primary B cells treated with LPS and IL-4 for 3 days were stained with PI to detect the cell viability. The B cells

Sample preparation	treated with LPS and IL-4 for 3 days were stained with Mito-Tracker Green (Yeasen) for observing mitochondrial mass and Mito-Tracker CMXRos (Yeasen) to measure the mitochondrial transmembrane potential.
Instrument	Aria 4 laser,Fortessa 5laser(BD BioSciences, San Jose, CA, USA)
Software	FACS Diva
Cell population abundance	5000-10000 cells were counted per sample.
Gating strategy	The predominant cell population on a FSC/SSC plot was gated,and was further selected by comparing FSC-H and FSC-W to identify single cells.

☒ Tick this box to confirm that a figure exemplifying the gating strategy is provided in the Supplementary Information.
